# Supervised biological network alignment with graph neural networks

**DOI:** 10.1093/bioinformatics/btad241

**Published:** 2023-06-30

**Authors:** Kerr Ding, Sheng Wang, Yunan Luo

**Affiliations:** School of Computational Science and Engineering, Georgia Institute of Technology, Atlanta, GA 30332, United States; Paul G. Allen School of Computer Science & Engineering, University of Washington, Seattle, WA 98195, United States; School of Computational Science and Engineering, Georgia Institute of Technology, Atlanta, GA 30332, United States

## Abstract

**Motivation:**

Despite the advances in sequencing technology, massive proteins with known sequences remain functionally unannotated. Biological network alignment (NA), which aims to find the node correspondence between species’ protein–protein interaction (PPI) networks, has been a popular strategy to uncover missing annotations by transferring functional knowledge across species. Traditional NA methods assumed that topologically similar proteins in PPIs are functionally similar. However, it was recently reported that functionally unrelated proteins can be as topologically similar as functionally related pairs, and a new data-driven or supervised NA paradigm has been proposed, which uses protein function data to discern which topological features correspond to functional relatedness.

**Results:**

Here, we propose GraNA, a deep learning framework for the supervised NA paradigm for the pairwise NA problem. Employing graph neural networks, GraNA utilizes within-network interactions and across-network anchor links for learning protein representations and predicting functional correspondence between across-species proteins. A major strength of GraNA is its flexibility to integrate multi-faceted non-functional relationship data, such as sequence similarity and ortholog relationships, as anchor links to guide the mapping of functionally related proteins across species. Evaluating GraNA on a benchmark dataset composed of several NA tasks between different pairs of species, we observed that GraNA accurately predicted the functional relatedness of proteins and robustly transferred functional annotations across species, outperforming a number of existing NA methods. When applied to a case study on a humanized yeast network, GraNA also successfully discovered functionally replaceable human–yeast protein pairs that were documented in previous studies.

**Availability and implementation:**

The code of GraNA is available at https://github.com/luo-group/GraNA.

## 1 Introduction

In biomedical research, it is often challenging or infeasible to directly perform experiments on humans due to technical or ethical reasons (O’Neil et al. 2017). Model organisms thus have been indispensable tools for studying fundamental questions in human disease and clinical applications. Compared to humans, model organisms are simpler biological systems for comprehensive function characterization, have faster generation cycles that facilitate genetic screens, and can be readily manipulated genetically (Irion and Nüsslein-Volhard 2022). The characterization and understanding of model organisms can provide great opportunities for translational studies in biomedicine. For example, baker’s yeast (*Saccharomyces cerevisiae*) has been used as the model organism to map molecular pathways of Parkinson’s disease in humans (Khurana et al. 2017).

A pivotal challenge to fully realizing the potential of model organism studies for studying biomedicine is transferring the functional knowledge we learned in one species to better understand the functions of the proteins from different species (Park et al. 2013). A popular strategy to find functionally similar proteins is through sequence similarity search (e.g. by BLAST; Altschul et al. 1990), yet sequence-similar proteins may perform different functions. In fact, it has been found that 42% of human-yeast orthologs are not functionally related (Balakrishnan et al. 2012; Gu and Milenković 2021), i.e. not sharing common functional annotations. Moreover, proteins perform functions by interacting with other proteins, which form biological pathways and protein–protein interaction (PPI) networks. Therefore, in many species, similar functions can be carried out by proteins that do not have the most similar sequences but instead have similar functional roles in a biological pathway (Park et al. 2013). For this reason, network alignment (NA) has emerged as a complementary solution to sequence alignment for identifying the functional correspondence of proteins of different species.

Traditionally, NA aims to find the node mapping between compared networks that can reveal topologically similar regions, rather than just similar sequences. This problem is closely related to the subgraph isomorphism problem of determining whether a network is a subgraph of the other (Ullmann 1976), which is known as NP-hard (Cook 1971). NA of biological networks has been widely studied in bioinformatics and a large number of NA methods have been developed. Those methods circumvented the intractable complexity of the isomorphism problem by heuristically defining topological similarity based on a node’s neighborhood structure. Examples include search algorithms (Patro and Kingsford 2012; Mamano and Hayes 2017), genetic algorithms (Saraph and Milenković 2014; Vijayan and Milenković 2017), random walk-based methods (Singh et al. 2008; Kalecky and Cho 2018), graphlet-based methods (Milenković et al. 2010; Malod-Dognin and Pržulj 2015), latent embedding methods (Fan et al. 2019; Li et al. 2022), and many others (Meng et al. 2016). Moreover, in the context of the NA of social networks, graph representation learning-based methods have been proposed (Chen et al. 2020).

Although based on various heuristics, most existing NA methods for biological networks have a common key assumption: proteins that are in similar topological positions with respect to other proteins in the PPI network tend to have the same functions. However, observations in recent studies questioned this assumption, in which nodes aligned by those methods, while having high topological similarity, did not correspond to proteins that perform the same functions, and the topological similarity of functionally related nodes was barely higher than that of functionally unrelated pairs (Elmsallati et al. 2015; Meng et al. 2016; Guzzi and Milenković 2018). The major reason for the failure of the assumption stems from the intrinsic noisy and incomplete nature of biological networks which contain a copious amount of spurious and missing edges. Even if we could obtain error-free PPI networks, the similar topology of cross-species subnetworks that share similar functions can be altered during evolution due to events such as gene duplication, deletion, and mutation. Therefore, solely relying on topological similarity to align biological networks may result in unsatisfactory accuracy.

Recently, Gu and Milenković (2020, 2021) proposed a new paradigm called data-driven NA to address the limitation of traditional NA methods. Essentially, this new paradigm transforms NA from an unsupervised problem to a supervised task, and supervised models are trained on *both* PPI network and protein function data to learn to align functionally similar nodes. The key insight is that, using function data as supervision, the model will be driven to tease topological features that are more informative for NA (termed as topological relatedness in Gu and Milenković 2020) apart from other signals, such as network noise or incompleteness that are likely to break the common assumption of traditional NA methods. In contrast, most traditional NA methods are unsupervised and may not easily capture such topological features. Gu *et al.* have developed supervised NA methods TARA and TARA++ (Gu and Milenković 2020, 2021), which first built graphlet features (Milenković and Pržulj 2008) of network nodes and trained a logistic classifier with function data to distinguish between functionally related and unrelated node pairs. While outperforming traditional unsupervised NA methods, TARA (or TARA++) still has several limitations. First, its prediction performance is suboptimal as the linear logistic classifier may not be able to capture high-order, non-linear topological features. In addition, TARA(++) is a two-stage method, where protein representations are learned in the first stage using unsupervised algorithms such as graphlet (Milenković et al. 2010) or node2vec (Grover and Leskovec 2016), and in the second stage NA based on the learned representations is performed using supervised logistic regression. The two-stage approach may result in suboptimal alignment quality as the representation learning in the first stage is not optimized toward maximizing the alignment accuracy. Moreover, TARA(++) is not readily extended from pairwise NA to other NA problems, such as heterogeneous NA and temporal NA, for large-scale networks due to the high computational cost of counting heterogeneous or temporal graphlets (Gu et al. 2018; Vijayan and Milenković 2018).

In this work, we develop GraNA, a more powerful and flexible supervised NA model for the data-driven NA paradigm for the pairwise, many-to-many NA problem (Guzzi and Milenković 2018). GraNA is a graph neural network (GNN) that learns informative representations for protein nodes and predicts the functionally related node pairs across networks in an end-to-end fashion. Following TARA-TS (Gu and Milenković 2021), GraNA also represents the two PPI networks to be aligned as a joint graph and integrates heterogeneous information as anchor links to guide the NA. As protein orthologs, defined as proteins/genes in different species that originated from the same ancestor, tend to retain function over evolution, GraNA further integrates across-network orthologous relationships as anchor edges to guide the alignment. One strength of GraNA is that heterogeneous data can be readily incorporated as additional nodes, edges, or features to facilitate NA. For example, GraNA integrates sequence similarity edges as additional anchor links to guide the alignment and pre-computed network embeddings as node features to better encode the topological roles of network nodes. GraNA is trained as a link prediction model, where function data (i.e. whether a given pair of proteins have functions in common) is used as training data. We also proposed a negative sampling strategy to improve the model training effectiveness. Since multi-modal data are integrated, GraNA is able to learn informative protein representations that reflect orthologous relationships, topology, and sequence similarity to better characterize functional similarity between proteins. Evaluated on NA tasks between five species, GraNA accurately aligned across-species protein pairs that are functionally similar. We further showed that the alignments produced by GraNA can be used to achieve accurate across-species protein function annotations. Moreover, we demonstrated GraNA’s applicability by applying it to predict the functional replacement of essential yeast genes by their human orthologs, in which GraNA re-discovered previously validated replaceable pairs in important pathways.

## 2 Methods

### 2.1 Problem formulation

In this article, we focus on pairwise NA of two species’ PPI networks. We are given as input an integrated graph $G=(G_{1},G_{2},E_{12})$, where the undirected graph $G_{k}=(V_{k},E_{k})$ is the PPI network of species *k* (*k *=* *1, 2), with *V_k_* as the set of the proteins and *E_k_* as the set of physical interactions between proteins; $E_{12}\subseteq V_{1}\times V_{2}$ is a set of across-network edges that serve as anchor links for aligning the PPIs, such as orthologous proteins pairs. In the data-driven NA framework, the NA problem is formulated as a supervised link prediction task, where a set of functionally related protein pairs $R=\{(u_{1},u_{2})|u_{1}\in V_{1},u_{2}\in V_{2}\}$ is given as training data to train a model to predict whether a new pair of proteins are functionally related. Following previous work (Gu and Milenković 2021; Li et al. 2022), the functional relatedness of two proteins is defined based on whether they share the same Gene Ontology (GO) terms (Section 3.1).

### 2.2 Overview of GraNA

**Figure 1.**
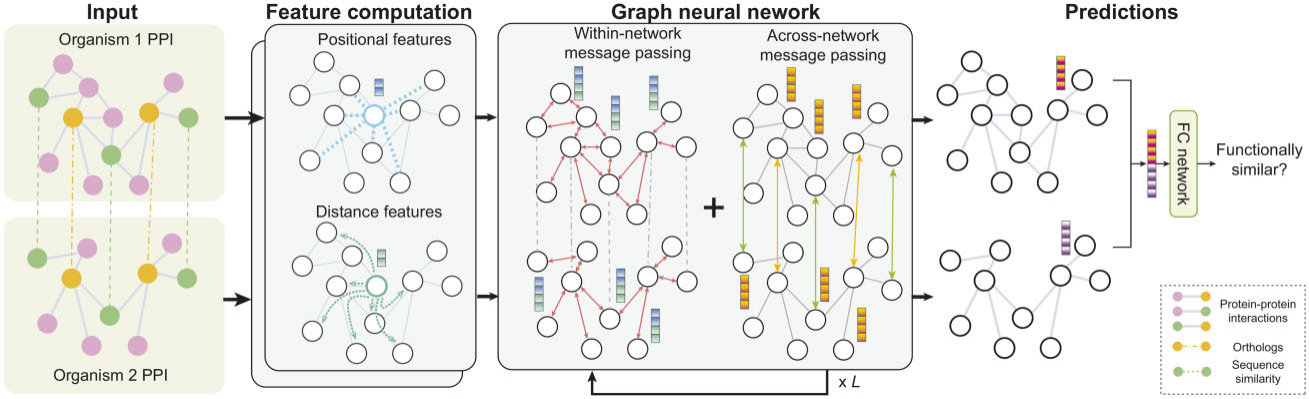
Schematic overview of GraNA. GraNA is a supervised GNN that aligns functionally similar proteins in the PPI networks of two species. It integrates orthologs and sequence similarity relationships as anchor links to guide alignments. GraNA derives positional and distance embeddings as node features for proteins and performs iterative within- and across-network message passing to learn protein representations that capture protein functional similarity. The concatenated representations of a pair of proteins are used to make the final prediction using a fully connected (FC) neural network.

We propose, GraNA, a novel framework based on GNNs for supervised NA (Fig. 1). Receiving PPIs *G*_1_, *G*_2_, and anchor links *E*_12_ as input, GraNA first builds positional and distance embeddings as node features for every node. It then uses a GNN, which performs both within- and across-network message passing through PPI edges and anchor links (orthologs and sequence similarity), to enhance and refine those features into final representations that capture topological and evolutionary similarity relationships. GraNA is trained with protein function data to predict whether a pair of across-network proteins share the same function.

### 2.3 GNN architecture of GraNA

GNNs have been widely used to model graph-structured data such as social networks, physical systems, or chemical molecules (Dwivedi et al. 2020). Here, we develop a novel GNN architecture, adapted from the Generalized Aggregation Network (Li et al. 2020a), to model our input PPI networks $G=(G_{1}=(V_{1},E_{1}),G_{2}=(V_{2},E_{2}),E_{12})$. The key of GNNs is the graph convolution (also known as message passing) where a node first aggregates the features from its neighbor nodes, updates them with neural network layers, and then sends out the updated features to its neighbors. Through iterative graph convolutions on the PPI networks *G*_1_, *G*_2_, and anchor links *E*_12_, our model can learn an embedding for each node that encodes information of both graph topology and relationships of anchor links. GraNA has *L* layers of graph convolution blocks, where the ℓ-th block contains a series of non-linear neural network layers that transform node *i*’s embedding $h_{i}^{\ell }\in R^{d}$ to $h_{i}^{\ell +1}\in R^{d}$, where ℓ∈[L] and $i\in [n],\;n=|V_{1}|+|V_{2}|$. In particular, $h_{i}^{0}$ is the initialized node feature (described in Section 2.4).

Within each graph convolution blocks are within- and across-network propagation layers that update the node embeddings. In the ℓ-th block, the node embeddings $h_{i}^{\ell }$ are first updated by the propagation along PPI edges (within-network message passing), in which a node aggregates its neighbor’s features using the attention mechanism: where $N^{\text{in}}(i)$ is the set of neighboring nodes of node *i* in terms of the within-network edge set $E_{1}\cup E_{2}$, and *γ* is a learnable parameter known as the inverse temperature. Next, node embeddings are updated with across-network message passing through anchor links: where $N^{\text{ac}}(i)$ is the set of neighboring nodes of node *i* in terms of the across-network edge set *E*_12_, and *ω* is a learnable temperature parameter. The final updated embedding $h_{i}^{\ell +1}$ is obtained using multi-layer perceptron (MLP) f,f′ followed by a residual connection (He et al. 2016): $h_{i}^{\ell +1}=f(h_{i}^{\ell }+m_{i})+f\prime (h_{i}^{\ell }+\mu_{i})+h_{i}^{\ell }$. In GraNA, we stacked seven graph convolution blocks to build the GNN, where each block performs one iteration of within-network propagation (Equation (1)) and one iteration of across-network propagation (Equation (2)). Pair normalization (Zhao and Akoglu 2019) and ReLU non-linear transformation (Nair and Hinton 2010) are applied between two adjacent convolution blocks.


(1) $$m_{i}=\sum_{j\in N^{\text{in}}(i)}\alpha_{ij}\cdot h_{j}^{\ell }\;\text{where}\;\alpha_{ij}=\frac{\;\text{exp}(\gamma \cdot h_{j}^{\ell })}{\sum_{j\prime \in N^{\text{in}}(i)}\;\text{exp}\;(\gamma \cdot h_{j\prime }^{\ell })},$$

(2) $$\mu_{i}=\sum_{j\in N^{\text{ac}}(i)}\beta_{ij}\cdot h_{j}^{\ell }\;\text{where}\;\beta_{ij}=\frac{\;\text{exp}(\omega \cdot h_{j}^{\ell })}{\sum_{j\prime \in N^{\text{ac}}(i)}\;\text{exp}(\omega \cdot h_{j\prime }^{\ell })},$$

After the graph convolution, the representations of nodes $i\in V_{1}$ and $j\in V_{2},\;h_{i}^{L}$ and $h_{j}^{L}$, are concatenated and passed to a two-layer MLP to out a probability score that predicts whether the two nodes should be aligned. GraNA is trained using the binary cross entropy loss. The hyperparameters for training GraNA were selected based on GraNA’s performances on valid sets, and we further tested the effect of different hyperparameters on GraNA’s performances. The details of how we select our hyperparameters, the effects different hyperparameters have upon GraNA, and other implementation details are provided in the Supplementary Information.

### 2.4 Network features of proteins

While GNNs are able to learn node embeddings that encode topological information of the input PPI network structure, previous studies have found that GNNs might perform poorly when the graph exhibit symmetries in local structure, such as node or edge isomorphism. This is related to the theoretic limitation of GNNs due to their equivalence to the 1-Weisfeiler-Lehman test of graph isomorphism (Xu et al. 2018). Some existing NA methods also suffered from this limitation. For example, the state-of-the-art NA method ETNA (Li et al. 2022) has to filter out nodes with the same neighborhood structure, since these are indistinguishable to their model when only topological information is used.

Inspired by several solutions in graph machine learning (Dwivedi et al. 2020; Li et al. 2020b), we introduce two types of node features, as the initializations of node embeddings $h_{i}^{0}$, to improve the expressiveness of our GNN model and facilitate the topological feature learning. We use two complementary network features, namely the graph Laplacian positional embeddings, which encode a node’s ‘position’ with respect to other nodes in the network, and the diffusion-based embeddings, which capture a node’s ‘distance’ to other nodes in random walks. Intuitively, the two types of embeddings capture the long-range relationships between network nodes. In GraNA, these features are incorporated as the initialization of node embeddings $h_{i}^{0}$ and then refined by message passing around each node’s direct neighbor vicinity. Therefore, GraNA can capture both local and global topological proximity in the network. Next, we describe how to construct the positional and distance features.

#### 2.4.1 Distance embeddings

Random walk or PageRank-based algorithms have been widely used to learn network embeddings (Perozzi et al. 2014; Grover and Leskovec 2016) and improve expressiveness of GNNs (Li et al. 2020b). For example, the distance matrix at the equilibrium states of a random walk with restart has been used to encode the topological roles of genes or proteins in molecular networks (Cho et al. 2016; Cowen et al. 2017). Following those ideas, in this work, we compute distance embeddings for network nodes using NetMF (Qiu et al. 2018), a unified framework that generalizes several previous network embedding methods (Perozzi et al. 2014; Grover and Leskovec 2016) and estimates the distance similarity matrix *M* in a closed form: where *A* is the *n *×* n* adjacency matrix of the network, *D* is the diagonal degree matrix, $\text{vol}(G)=\sum_{i=1}^{n}D_{ii}$ is the volume of the graph *G*, *b* is the parameter for negative sampling, *T* is the context window size. Unlike the adjacency matrix *A* that only contains direct neighbor relationships, the NetMF matrix *M* encodes the similarity between long-distance neighbors. The entry *M_ij_* approximates the number of paths with length up to *T* between nodes *i* and *j*. In GraNA, setting *b *=* *1 and *T *=* *10 following the default choices (Qiu et al. 2018), we computed matrices *M* for the two input PPIs separately and used the *i*-th row of log M as the distance embedding for node *i*. As the row vector has a high dimension as the number of nodes, we applied a linear neural network layer to project the row vector from dimension *n* to *d* (d≪n), where *d* is the hidden dimension in GraNA’s graph convolution layers.


(3) $$M=\frac{\text{vol}(G)}{bT}\left(\sum_{r=1}^{T}{\left(D^{-1}A\right)}^{r}\right)D^{-1},$$

#### 2.4.2 Positional embeddings

In addition to distance embeddings, we further build positional features such that nodes nearby in the network have similar embeddings while distant nodes have different embeddings. For this purpose, GraNA applies the Laplacian positional encoding, which has been shown to be able to encode graph positional features in GNNs (Dwivedi et al. 2020). The idea is to use graph Laplacian eigenvectors that embed the graph into Euclidean space while preserving the global graph structure. Mathematically, the normalized graph Laplacian is factorized as $L=I-D^{-1/2}AD^{-1/2}=U^{⊤}\Lambda U$, where Λ and *U* refers to the eigenvalues and Laplacian eigenvectors, respectively. In GraNA, the *d*-smallest non-trivial eigenvectors are used as the positional embeddings and concatenated with the distance embeddings together as the initialized node features $h_{i}^{0}$.

### 2.5 Integrating heterogeneous information for NA

In addition to PPIs, there are other types of relationships that can help characterize the functional similarity of proteins, such as gene–gene interactions, sequence similarity, phenotype similarity, and associations between proteins and other entities such as diseases. A naive way to integrate multiple data sources is to collapse them as additional but the same type of nodes and edges in a flattened network, which, however, may lose context-specific information. Heterogeneous data integration, which treats distinct types of nodes and edges separately, has been shown effective to integrate diverse data sources (Cho et al. 2016; Luo et al. 2017). A few previous NA studies consider the heterogeneous NA problem, but their approaches required non-trivial modifications in the optimization objective and feature engineering as compared to the homogeneous NA problem. On the contrary, one of the major advantages of GraNA is that it can readily integrate heterogeneous information to facilitate the alignment of networks by simply including the data as additional nodes, edges, or feature embeddings and applying heterogeneous graph convolutions to capture context-specific information.

As a proof-of-concept, here we apply GraNA to incorporate sequence similarity relationships as another type of anchor links in addition to the orthologous relationships. Now we have two sets of across-network edges as input, which are denoted as $E_{12}^{\left(r\right)}$ for *r *=* *1, 2. To learn embeddings from heterogeneous data, we perform separate across-network message passing for each edge type: $\mu_{i}^{\left(r\right)}=\sum_{j\in N_{r}^{\text{ac}}(i)}\beta_{ij}\cdot h_{j}^{\ell }$. Compared to Equation (2), note that the aggregation and the weights *β_ij_* here are defined on *i*’s neighbor nodes that are connected by the *r*-th type of edges $N_{r}^{\text{ac}}(i)$, instead of all neighbors N(i). After performing both types of message passing, we obtain the updated node embedding using a sum pooling operation over all edge types: $h_{i}^{\ell +1}=f(h_{i}^{\ell }+m_{i})+\sum_{r}f^{\left(r\right)}(h_{i}^{\ell }+\mu_{i}^{\left(r\right)})+h_{i}^{\ell }$, where $f^{\left(r\right)}$ is a fully connected neural network specific to edge type *r*. We expect that, by multi-view information from orthologs and sequence similarity edges, GraNA can better distill the topological features that are useful to predict functional relatedness. Of note, GraNA is a generic framework, and other types of node or edge data can be integrated into GraNA in a similar way.

### 2.6 Enhancing model learning with hard negative sampling

Supervised NA essentially is a positive-unlabeled learning problem, meaning that we only observed positive protein pairs that are functionally related (e.g. have at least one GO term in common), denoted as $I_{p}=\{(p,q)|\text{proteins}\;p\;\text{and}\;q\;\text{are functionally related}\}$, without observing validated negative samples. For a new pair $(p^{*},q^{*})\notin I_{p}$, it does not necessarily mean that the two proteins do not have the same function, rather, it is more likely their functions have not been thoroughly characterized by experiments. To generate negative samples for training a supervised classifier to distinguish functionally related and unrelated pairs, previous NA methods usually chose to randomly sample a set of pairs not in $I_{p}$ as the negative set $I_{n}$ (Gu and Milenković 2020, 2021).

We reason that the random negative sampling might lead the machine learning model to learn the node’s presence in the training data rather than functional relatedness. Denote $V_{1}^{p}$ as the set of proteins in PPI *G*_1_ that are involved in positive pairs, i.e. $V_{1}^{p}=\{p|p\in V_{1}\;\text{and}\;(p,q)\in I_{p}\;\text{for some}\;q\in V_{2}\}$, and $V_{2}^{p}$ has a similar meaning. As only a small fraction of proteins in *V*_1_ and *V*_2_ are involved in the positive set, for most randomly sampled negative pairs (*p*, *q*), it is likely that *p* and *q* are new proteins that did not occur in $V_{1}^{p}$ or $V_{2}^{p}$, respectively. Due to the distribution discrepancy between positive and negative samples in the training set, machine learning models trained on this data may only learn to predict for a given pair of proteins (*p*, *q*) whether $p\overset{?}{\in }V_{1}^{p}$ or $q\overset{?}{\in }V_{2}^{p}$, rather than predicting $(p,q)\overset{?}{\in }I_{p}$.

To encourage the model to learn the functional relatedness instead of node representativeness in the training data, we propose a hard negative sampling strategy to construct the negative set, where the sampled negative edges must contain nodes that have both appeared in positive edges. We achieve this by performing edge swap between positive edges: given two positive pairs (*p*_1_, *q*_1_) and (*p*_2_, *q*_2_), we swap their endpoints and add new edges (*p*_1_, *q*_2_) and (*p*_2_, *q*_1_) to the negative set if there did not show in $I_{p}$. Equivalently, the set of negative edges is defined as $I_{n}^{\text{hard}}=\{(p,q)|p\in V_{1}^{p},q\in V_{2}^{p},(p,q)\notin I_{p}\}$. In our experiments, we also compared to two other negative sampling strategies, including the “easy” sampling used in previous NA studies (Gu and Milenković 2020, 2021): $I_{n}^{\text{easy}}=\{(p,q)|p\in V_{1},q\in V_{2},(p,q)\notin I_{p}\}$, and a “semi-hard” sampling that requires a sampled negative edge to contain at least one node that has appeared in positive edges: $I_{n}^{\text{semi}}=\{(p,q)|(p\in V_{1}^{p}\;\text{and}\;q\in V_{2})\;\text{or}\;(p\in V_{1}\;\text{and}\;q\in V_{2}^{p}),(p,q)\notin I_{p}\}$.

## 3 Results

We performed several experiments to assess GraNA’s ability to capture the functional similarity of proteins and predict protein functions across species. We also conducted ablation studies to better understand the model’s prediction performance. Furthermore, we used a proof-of-concept case study to demonstrate GraNA’s applicability in functional genomics.

### 3.1 Datasets

#### 3.1.1 Network data

The PPI network data of six species (*S. cerevisiae*, *Schizosaccharomyces pombe*, *Homo sapiens*, *Caenorhabditis elegans*, *Mus musculus*, and *Drosophila melanogaster*) were downloaded from BioGRID (version 3.5.187) (Stark et al. 2006). We used both orthologs and sequence similarity relationships as anchor links to guide NA. For orthologs, we followed the ETNA study (Li et al. 2022) and downloaded orthology data from OrthoMCL (version 6.1) (Li et al. 2003). For sequence-similar pairs, we retrieved the expert-reviewed sequences, if any, of proteins in our PPI networks from the UniProtKB/Swiss-Prot database (Consortium 2023). We then used MMseqs2 (Steinegger and Söding 2017) to perform sequence similarity searches between the proteins of pairwise species and kept protein pairs with an E-value $\leq 10^{-7}$ as anchor links. We chose this cutoff following previous work (Kalecky and Cho 2018; Gu and Milenković 2021), and we observed that varying this cutoff in a wide range had no significant impact on our GraNA’s prediction performance (Supplementary Fig. S1). The statistics of the PPI networks and the anchor links can be found in Supplementary Tables S1 and S2.

#### 3.1.2 Functional annotations

We collected the functional annotations (terms) from GO (Ashburner et al. 2000) (2020-07-16) and considered two proteins to be functionally similar if their corresponding genes have the same GO terms. Following ETNA (Li et al. 2022), we only kept annotations related to the Biological Process category, which are propagated through *is a* and *part of* relations, and included evidence codes EXP, IDA, IMP, IGI, and IEP. As GO terms appearing at the higher levels of the GO hierarchy might be too general or redundant, following ETNA (Li et al. 2022) and other studies (Gu and Milenković 2020, 2021), we focused our analyses on specific functions by creating a slim set of GO terms associated with at least 10 genes but no more than 100 genes. Other expert-curated GO slim terms were also added to this slim set (Greene et al. 2015). The statistics of functionally similar protein pairs between species can be found in Supplementary Table S2.

### 3.2 GraNA better exploits topological similarity for NA

We first assessed GraNA’s ability for NA by applying it to align the networks between human and four major model organisms, including *S. cerevisiae*, *M. Musculus*, *C. elegans*, and *D. melanogaster*, and between two yeast species (*S. cerevisiae* and *S. pombe*). The prediction task was formulated as a link prediction problem, i.e. predicting whether two proteins have the same function. We created an out-of-distribution train/test split (with a ratio of 8:2) such that proteins present in the training set never occur in the test set. In another more challenging split, we further forced that the training proteins and test proteins do not have >30% sequence identity.

We compared GraNA to two unsupervised embedding-based methods (ETNA (Li et al. 2022), MUNK (Lim et al. 2018)), a graph theoretic method (IsoRank (Singh et al. 2008)), a sequence similarity-based method (MMseqs2 (Steinegger and Söding 2017)), and two supervised methods (TARA-TS and TARA++ (Gu and Milenković 2021)). We used the same PPI networks and orthologs anchor links for all baseline methods. Anchor links for protein pairs that share GO terms were removed to avoid data leakage. To make a fair comparison, we included a variant of our method (GraNA-o) that only used orthologs (without sequence-similar pairs) as anchor links. Unsupervised methods were evaluated on the same test set used for supervised methods. The running time analyses of GraNA and representative baseline methods can be found in Supplementary Table S6.

**Figure 2.**
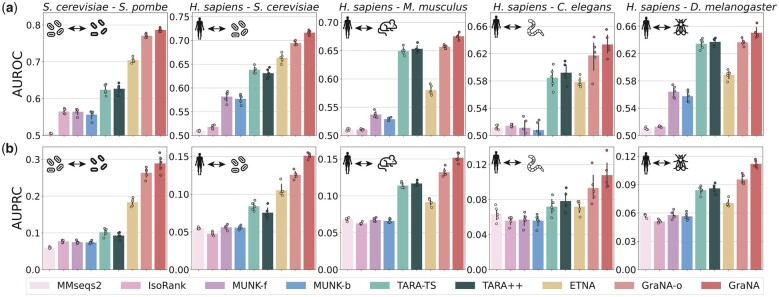
Performances of NA prediction. GraNA and other baselines were evaluated for aligning functionally similar proteins across five pairs of species, using (a) AUROC and (b) AUPRC as metrics. GraNA-o is a variant of GraNA that only uses orthologs as anchor links whereas GraNA refers to the full model that uses both orthologs and sequence similarity as anchor links. The default E-value cutoff $10^{-3}$ is used for MMseqs2. As MUNK is not a bidirectional NA method, the performances of its forward and backward predictions were shown separately as MUNK-f and MUNK-b. Performances were evaluated using five independent train/test data splits. Raw AUROC and AUPRC scores are provided in Supplementary Tables S4 and S5.

The evaluation results suggested that GraNA consistently outperformed other methods for aligning functionally related proteins in all five NA tasks in terms of the AUROC and AUPRC metrics (Fig. 2). Precision, recall, and total number of predicted alignments were reported in Supplementary Figs. S11, S12, and S13. We first confirmed the advantage of the supervised NA paradigm over the traditional unsupervised paradigm: GraNA(-o) substantially improved other unsupervised methods (MMseqs2, IsoRank, MUNK, and ETNA) with clear margins. For example, the AUROC and AUPRC improvements achieved by GraNA over the best unsupervised method (ETNA) were 11% and 55%, respectively (averaged over five tasks). Compared to those unsupervised methods that entirely rely on the topology to align nodes and are susceptible to the noise and incompleteness in biological networks, GraNA further leveraged function data as direct supervision signals to tease topological features that are directly related functional relatedness from background noise and greatly improved the alignment quality.

In addition, compared to TARA-TS and TARA++, the only methods for the supervised NA paradigm in literature, we found that our method is a more powerful deep learning solution for supervised NA. For example, GraNA-o on average had 53% higher AUPRC scores than TARA-TS. Interestingly, TARA-TS, despite as a supervised method, sometimes even had a lower performance than the state-of-the-art unsupervised method ETNA. The potential reason is that TARA-TS only used a linear logistic model that only able to model linear feature interactions in the data, while GraNA is an end-to-end GNN, which captures more complex, non-linear feature dependencies, and can better exploit topological similarity and predict node alignment.

Moreover, although GraNA-o outperformed other methods in most scenarios, in a few cases, it was only on par with the second-best baseline (TARA-TS; Fig. 2, 3rd and 5th columns). However, we found that when integrating both orthologs and sequence similarity as anchor links, the full model (GraNA) further improved GraNA-o and outperformed all other baselines in all tasks in both AUROC and AUPRC, suggesting that GraNA was an effective tool to integrate heterogeneous data for boosting the NA performance. In contrast, we observed that TARA-TS, even when given the two types of anchor links, was not able to improve the alignment performance (to be discussed in Section 3.4 and Fig. 4a).

On a more challenging data split where the sequences in the train and test sets have no sequence identity >30%, we also observed that GraNA clearly outperformed the second best baselines ETNA and TARA-TS (Supplementary Figs. S3 and S4). We also had similar observations when using other sequence identity cutoffs to create the train/test splits (Supplementary Fig. S2). This strict benchmark suggested that GraNA can generalize its prediction for proteins that are sequence-dissimilar from what it has seen in the training data.

Additionally, we created another challenging evaluation dataset based on a temporary split strategy, where the snapshot of the GO database as of 2018-07-02 was used as training data, and the GO snapshot as of 2022-12-04, excluding all training annotations, was used as test data. On this dataset, we again observed similar results where GraNA outperformed baselines such as ETNA and TARA-TS (Supplementary Fig. S5). This demonstrated GraNA’s generalizability when making predictions for proteins whose functions are not completely characterized.

Overall, these results demonstrated that GraNA can better explore topological similarity to accurately align networks. The flexible GNN framework further allowed GraNA to integrate heterogeneous data types that capture multi-view similarity relationships to improve the alignment quality.

### 3.3 GraNA translates accurate NAs to function predictions

One important application of NA is to better understand human protein functions by transferring our learned function knowledge about model organisms. Therefore, after evaluating the performance of aligning functionally related proteins, we next studied whether the NAs produced by GraNA can facilitate protein function prediction. Here, we applied GraNA to generate the alignments between humans and the four model organisms. Then, we considered the top 5,000 ranked protein pairs aligned by GraNA and calculate the Jaccard index between the functional annotations of the two proteins in each pair. As a protein may have multiple functions, this evaluation aimed to quantify the overlap between the sets of functions of the two aligned proteins, which was more complex and challenging than the evaluation in the last section which predicted whether two proteins share at least one function. We also compared a random baseline that randomly samples 5000 pairs from proteins that have at least one GO term, in addition to our previously introduced baselines. Furthermore, we have also evaluated GraNA in an established protein function prediction framework (Meng et al. 2016).

**Figure 3.**
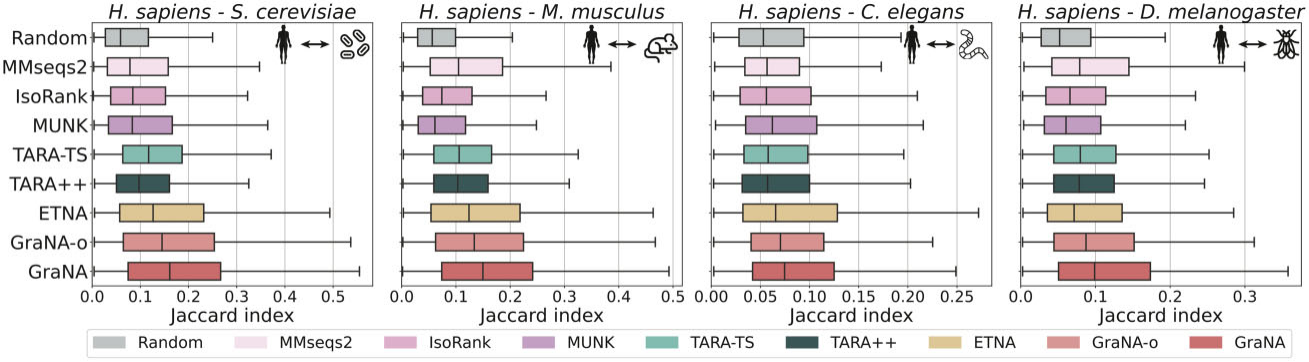
Performance of protein function prediction. Based on the NAs produced by each method for four pairs of species (*H. sapiens*–*S. cerevisiae*, *H. sapiens*–*M. Musculus*, *H. sapiens*–*C. elegans*, *H. sapiens*–*D. melanogaster*), we chose the top 5000 ranked protein pairs and transferred all the functional annotations of one protein in an aligned pair to predict the other protein’s function. The accuracy of the function prediction was evaluated by calculating the Jaccard index between the sets of the two aligned proteins. Box plots showed the distribution of the Jaccard index of the top 5000 aligned pairs for each method on five NA tasks.

We observed from Fig. 3 that, even with the partial model GraNA-o, our method has already outperformed other methods on three out of the four tasks in terms of Jaccard similarity. The full model GraNA, which integrated heterogeneous orthologs and sequence similarity edges, further boosted the function prediction performance. These results suggested that GraNA was able to not only align functionally similar protein pairs but also prioritize “most similar” pairs to the top of its prediction list. GraNA’s ability to prioritize functionally similar proteins has important implications when studying human diseases, since it can suggest the most functionally similar counterpart of a human gene in model organisms for detailed characterization. Moreover, we noted that the improvements achieved by GraNA over other methods were more pronounceable for species with high-quality PPI networks (e.g. *S. cerevisiae*). On the alignment task between human (*H. sapiens*) and roundworm (*C. elegans*), GraNA achieved performance on par with the second best baseline, which was likely due to that the PPI of *C. elegans* is the sparsest among all four model organisms (density <0.2%). This finding was consistent with the ETNA study (Li et al. 2022). We also compared GraNA with other methods using the functional coherence (FC) metric (Singh et al. 2008; Chindelevitch et al. 2013), a variant of the Jaccard index that only focuses on standardized GO terms to avoid bias caused by terms from different levels of the GO hierarchy, and observed similar performance (Supplementary Fig. S10). Additionally, by using the protein function prediction framework (Meng et al. 2016), we observed that GraNA predicted a smaller set of predictions with higher precision compared to TARA++, which is useful when high-confidence and limited false positive predictions are desired (Supplementary Fig. S14). Overall, this experiment here suggested that GraNA translated its effective NAs to the accurate predictions of protein functions, demonstrating its potential for across-species functional annotations.

### 3.4 Analyses of key model designs in GraNA

Having validated that GraNA outperformed state-of-the-art methods for aligning networks and predicting functions, we performed ablation studies to understand the GraNA model in more detail and attribute performance improvements to several key design choices in GraNA.

#### 3.4.1 Heterogeneous anchors

**Figure 4.**
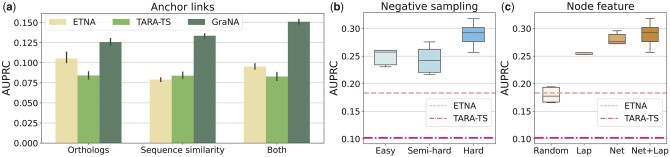
Ablation studies validated key designs of GraNA. (a) Comparison between GraNA and two of the best baselines, TARA-TS and ETNA on heterogeneous data integration, where either orthologs, sequence similarity, or both were used as the anchor links for the NA between *H. sapiens* and *S. cerevisiae*. (b–c) Ablation analyses that compared different negative sampling strategies (b) and node features (c) for the NA between *S. cerevisiae* and *S. pombe*. AUPRC scores of the two best baselines (ETNA and TARA-TS) were shown in (b) and (c) for reference. Performances were based on five independent trials of train/test split. Comparisons on all species can be found in Supplementary Information. (Lap: Laplacian embeddings; Net: NetMF embeddings.).

As GraNA is a flexible framework to integrate heterogeneous data, we first investigated the effects of using heterogeneous data on the performance of NA. We compared GraNA variants that used only orthologs, only sequence similarity, or both as anchor links. We observed that with either of the anchor links, GraNA was able to achieve an AUPRC better than the two best baselines (ETNA and TARA-TS) and combining both of them led to the best AUPRC score (Fig. 4a and Supplementary Fig. S6). Interestingly, we found the two baselines, when given two types of anchors, did not improve their NA accuracy compared to when a single type of anchor was used (Fig. 4a and Supplementary Fig. S7). These comparisons indicated that information contained in the two types of edges are not redundant but complementary, and GraNA can integrate them more effectively than other baselines. The major reason was that GraNA implemented separate message passing mechanisms to handle different types of anchors, while ETNA and TARA-TS (with node2vec features (Grover and Leskovec 2016)) mixed them as a single type of edges. We expect that integrating more data that capture multiple aspects of protein similarity can further help GraNA to better characterize protein functional relatedness.

#### 3.4.2 Hard negative sampling

Another novel design in GraNA is the hard negative sampling which prevented the model from only learning from trivial training samples. To better illustrate this, we compared GraNA models trained with three negative sampling strategies, including easy, semi-hard, and hard negative sampling (Methods). We observed that GraNA trained with easy and semi-hard samplings already outperformed the second-best baseline, and using the hard sampling further improved the AUPRC by 20% and showed a more significant margin over baselines (Fig. 4b). Hard negative sampling is a critical ingredient that makes GraNA accurate and generalizable. As discussed in the Methods section, random negative sampling tends to create a training set that confuses the machine learning model, and the model may just learn whether protein appeared in the training set rather than the functional relatedness between protein pairs. In contrast, hard negative sampling forces our model to discriminate between functionally related and unrelated pairs.

#### 3.4.3 Node features

We used both distance features (NetMF embeddings) and positional features (Laplacian embeddings) to initialize the node features in GraNA. Here, we analyse the effect of the node features by comparing GraNA variants that used only one or both of the NetMF and Laplacian embeddings, or randomly initialized node features. We observed that with random node features, the prediction performance was only comparable with the unsupervised ETNA method (Fig. 4c). When replacing the random features with network-informed features (NetMF and Laplacian), GraNA significantly improved its AUPRC scores. Finally, incorporating both embeddings led to the highest AUPRC score. This comparison underscored the effectiveness of using informative features. Although the GNN model alone was able to capture topological properties of network nodes, it still only captured localized information as a node’s features were only propagated to its nearby neighbors with a few times (e.g. <10) of message passing. However, the two embeddings we used were able to encode global, long-range neighbor relationships between nodes, which were complementary to the topological features learned by the GNN and jointly enhanced GraNA’s effectiveness.

### 3.5 Application: predicting replaceability for a humanized yeast network

Finally, we demonstrate the applicability of GraNA using a task of identifying replaceable human–yeast gene pairs. Recent studies have identified many human genes that can substitute for their yeast orthologs and sustain yeast growth (Kachroo et al. 2015; Laurent et al. 2020), which provides a tractable system known as “humanized yeast” to allow for high-throughput assays of human gene functions. Given that not all yeast genes can be replaced by their human orthologs, biological NA methods might become useful tools to predict the replaceability among human-yeast orthologs.

**Figure 5.**
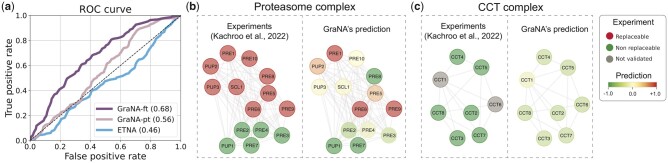
An application of GraNA on predicting replaceable human–yeast gene pairs in humanized yeast network. GraNA was used to predict whether human genes can replace their yeast orthologs for the functions in humanized yeast network. (a) Using 414 validated positive and negative human–yeast gene pairs by Kachroo et al. (2015) as the test set, we compared a pre-trained GraNA model (GraNA-pt), a fine-tuned GraNA model (GraNA-ft), and ETNA to evaluate their ability to distinguish replaceable and non-replaceable human-gene pairs in the test set. The performance of GraNA-ft was evaluated using 5-fold cross-validation whereas GraNA-pt and ETNA were evaluated on the whole test set directly. (b–c) Case studies where GraNA was used to predict the replaceability in two pathways: (b) Proteasome complex and (c) CCT complex. We visualized the complex validated by Kachroo et al. (2022) for comparison, where validated replaceable genes were colored in red, non-replaceable genes in green, and unvalidated genes in gray. For GraNA’s predicted network, the predicted score for each gene was normalized into a z-score and colored with a gradient colormap from green (most non-replaceable) to red (most replaceable).

We collected the experiment data from Kachroo et al. (2015), which has assayed 414 essential yeast genes for complementation by their human orthologs and found 47% of them could be humanized. After filtering out genes that are not included in the PPI network of *S. cerevisiae* that we used in this work, we obtained 411 gene pairs, out of which 174 replaceable pairs are labeled as positive samples and the remaining as negative. To avoid potential signal leakage, in our data we further removed 169 orthologs that coincide with the 411 pairs. Using this data as a binary classification test set, we first applied a baseline method, ETNA, to predict the replaceability of each human–yeast pair. We observed that ETNA’s predicted performance was nearly random (AUC ∼0.5; Fig. 5a). This was not surprising because, by design, ETNA was trained to classify between orthologs and non-orthologs, while all the positive and negative pairs in the test sets here are all human-yeast orthologs, which appeared to be indistinguishable to ETNA. Next, we applied the GraNA model pre-trained on our *H. sapiens*–*S. cerevisiae* alignment task (GraNA-pt) to predict for those 411 gene pairs. Even though GraNA-pt was not directly trained to predict replaceability, we found that it still had a better-than-random prediction accuracy (AUC = 0.56; Fig. 5a) on the test set, which suggested that the functional similarity relationships captured by GraNA were relatively more generalizable. After fine-tuning the trained GraNA model on the 411 gene pairs by re-training the parameter of the top MLP layers and freezing GNN layers, we observed that this model (GraNA-ft) reached an AUC of 0.68 in 5-fold cross-validation (Fig. 5a), which was higher than the AUC of the supervised TARA-TS model (Supplementary Fig. S8). This suggested that the prediction accuracy of GraNA on this task could be improved with direct supervision.

As a case study, we applied GraNA to study the replaceability in protein complexes. We selected as two examples the proteasome complex and the CCT complex that have experimental validation data (Kachroo et al. 2015, 2017, 2022). In both examples, we used the genes in the complex as the test set and the remaining genes with experimental validation data as the training set. For the Proteasome complex that contains both replaceable and non-replaceable genes, except for *PRE8*, GraNA correctly predicted a positive z-score for replaceable genes and a negative z-score for non-replaceable genes (AUC = 0.91; Fig. 5b). For the CCT complex that was mainly enriched with non-replaceable genes, GraNA’s prediction also recapitulated the replaceability in the network, where validated non-replaceable genes were predicted with a negative z-score (Fig. 5c).

Overall, these results demonstrated the applicability of GraNA for extending the NAs to empower other functional analyses of genes and proteins.

## 4 Conclusion

NA is a fundamental problem in various domains, such as linking users across social network platforms (Zafarani and Liu 2013), unifying entities across different knowledge databases (Zhu et al. 2017), and aligning keypoints in computer vision (Sarlin et al. 2020). In this article, we studied the NA problem for biological networks. We have presented GraNA, a deep learning model for aligning functionally related proteins in cross-species PPI networks. Our work was motivated by the recently proposed supervised NA methods such as TARA/TARA-TS (Gu and Milenković 2020, 2021), which represent the two PPIs being aligned as a joint graph connected by anchor links and integrate topology, sequence, and function information to characterize the function similarity between cross-species protein pairs. GraNA integrates PPI networks, ortholog and sequence similarity relationships, network distance and positional embeddings, and protein function data to learn to align across-species proteins that are functionally similar. Experiments showed that GraNA outperformed state-of-the-art NA methods, including both supervised and unsupervised approaches, on aligning pairwise PPI networks for five species, and the high-quality NAs of GraNA also enable accurate functional prediction across species. We further investigated several key model designs of GraNA that led to performance improvements and demonstrated the applicability of GraNA using a case study of predicting replaceability in humanized yeast network. GraNA is a flexible framework and can be readily extended in the future to integrate diverse types of entity and association data to facilitate NA. As previous methods such as TARA, GraNA can also be generalized to study other NA problems, including multi-species NA and temporary NA.

## Supplementary Material

btad241_Supplementary_Data

## Data Availability

The datasets underlying this article were derived from sources in the public domain, and we used the processed version of datasets from ETNA (Li et al. 2022): BioGRID: https://thebiogrid.org/. OrthoMCL: https://orthomcl.org/orthomcl/app/. UniProtKB: https://www.uniprot.org/. Gene Ontology (GO): http://geneontology.org/.

## References

[btad241-B1] Altschul SF , Gish W, Miller W et al Basic local alignment search tool. J Mol Biol 1990;215:403–10. 2231712 10.1016/S0022-2836(05)80360-2

[btad241-B2] Ashburner M , Ball CA, Blake JA et al Gene ontology: tool for the unification of biology. Nat Genet 2000;25:25–9. 10802651 10.1038/75556 PMC3037419

[btad241-B3] Balakrishnan R , Park J, Karra K et al Yeastmine-an integrated data warehouse for Saccharomyces cerevisiae data as a multipurpose tool-kit. Database 2012;2012:bar062. 22434830 10.1093/database/bar062 PMC3308152

[btad241-B4] Chen H , Yin H, Sun X et al Multi-level graph convolutional networks for cross-platform anchor link prediction. In: *Proceedings of the 26th ACM SIGKDD International Conference on Knowledge Discovery & Data Mining,* 2020. pp. 1503–11.

[btad241-B5] Chindelevitch L , Ma CY, Liao CS et al Optimizing a global alignment of protein interaction networks. Bioinformatics 2013;29:2765–73. 24048352 10.1093/bioinformatics/btt486 PMC3799479

[btad241-B6] Cho H , Berger B, Peng J et al Compact integration of multi-network topology for functional analysis of genes. Cell Syst 2016;3:540–8.e5. 27889536 10.1016/j.cels.2016.10.017 PMC5225290

[btad241-B7] Consortium TU Uniprot: the universal protein knowledgebase in 2023. Nucleic Acids Res 2023;51:D523–31. 36408920 10.1093/nar/gkac1052 PMC9825514

[btad241-B8] Cook SA. The complexity of theorem-proving procedures. In *Proceedings of the Third Annual ACM Symposium on Theory of Computing,* 1971. pp. 151–8.

[btad241-B9] Cowen L , Ideker T, Raphael BJ et al Network propagation: a universal amplifier of genetic associations. Nat Rev Genet 2017;18:551–62. 28607512 10.1038/nrg.2017.38

[btad241-B10] Dwivedi VP , Joshi CK, Luu AT et al Benchmarking graph neural networks. *Journal of Machine Learning Research 2023;* 24(43):1–48.

[btad241-B11] Elmsallati A , Clark C, Kalita J et al Global alignment of protein-protein interaction networks: a survey. IEEE/ACM Trans Comput Biol Bioinform 2016;13:689–705. 26336140 10.1109/TCBB.2015.2474391

[btad241-B12] Fan J , Cannistra A, Fried I et al Functional protein representations from biological networks enable diverse cross-species inference. Nucleic Acids Res 2019;47:e51. 30847485 10.1093/nar/gkz132 PMC6511848

[btad241-B13] Greene CS , Krishnan A, Wong AK et al Understanding multicellular function and disease with human tissue-specific networks. Nat Genet 2015;47:569–76. 25915600 10.1038/ng.3259 PMC4828725

[btad241-B14] Grover A , Leskovec J. node2vec: scalable feature learning for networks. In: *Proceedings of the 22nd ACM SIGKDD International Conference on Knowledge Discovery and Data Mining,* San Francisco, 2016. pp. 855–64. 10.1145/2939672.2939754 PMC5108654 27853626

[btad241-B15] Gu S , Milenković T. Data-driven network alignment. PLoS One 2020;15:e0234978. 10.1371/journal.pone.0234978. 32614833 PMC7331999

[btad241-B16] Gu S , Milenković T. Data-driven biological network alignment that uses topological, sequence, and functional information. BMC Bioinformatics 2021;22:1–24. 33514304 10.1186/s12859-021-03971-6 PMC7847157

[btad241-B17] Gu S , Johnson J, Faisal FE et al From homogeneous to heterogeneous network alignment via colored graphlets. Sci Rep 2018;8:1–16. 30131590 10.1038/s41598-018-30831-w PMC6104050

[btad241-B18] Guzzi PH , Milenković T. Survey of local and global biological network alignment: the need to reconcile the two sides of the same coin. Brief Bioinform 2018;19:472–81. 10.1093/bib/bbw132. 28062413

[btad241-B19] He K , Zhang X, Ren S et al Deep residual learning for image recognition. In: *Proceedings of the IEEE Conference on Computer Vision and Pattern Recognition*, 2016. pp. 770–8.

[btad241-B20] Irion U , Nüsslein-Volhard C. Developmental genetics with model organisms. Proc Natl Acad Sci USA 2022;119:e2122148119. 35858396 10.1073/pnas.2122148119 PMC9335277

[btad241-B21] Kachroo AH , Laurent JM, Yellman CM et al Systematic humanization of yeast genes reveals conserved functions and genetic modularity. Science 2015;348:921–5. 25999509 10.1126/science.aaa0769 PMC4718922

[btad241-B22] Kachroo AH , Laurent JM, Akhmetov A et al Systematic bacterialization of yeast genes identifies a near-universally swappable pathway. Elife 2017;6:e25093. 28661399 10.7554/eLife.25093 PMC5536947

[btad241-B23] Kachroo AH , Vandeloo M, Greco BM et al Humanized yeast to model human biology, disease and evolution. Dis Model Mech 2022;15:dmm049309. 35661208 10.1242/dmm.049309 PMC9194483

[btad241-B24] Kalecky K , Cho YR. Primalign: pagerank-inspired Markovian alignment for large biological networks. Bioinformatics 2018;34:i537–46. 29949962 10.1093/bioinformatics/bty288 PMC6022567

[btad241-B25] Khurana V , Peng J, Chung CY et al Genome-scale networks link neurodegenerative disease genes to *α*-synuclein through specific molecular pathways. Cell Syst 2017;4:157–70.e14. 28131822 10.1016/j.cels.2016.12.011 PMC5388136

[btad241-B26] Laurent JM , Garge RK, Teufel AI et al Humanization of yeast genes with multiple human orthologs reveals functional divergence between paralogs. PLoS Biol 2020;18:e3000627. 32421706 10.1371/journal.pbio.3000627 PMC7259792

[btad241-B27] Li G , Xiong C, Thabet A et al Deepergcn: all you need to train deeper gcns. *arXiv.* 2020a.

[btad241-B28] Li L , Stoeckert CJ, Roos DS et al Orthomcl: identification of ortholog groups for eukaryotic genomes. Genome Res 2003;13:2178–89. 12952885 10.1101/gr.1224503 PMC403725

[btad241-B29] Li L , Dannenfelser R, Zhu Y et al Joint embedding of biological networks for cross-species functional alignment. *bioRxiv.* 2022. 10.1093/bioinformatics/btad529 PMC10477935 37632792

[btad241-B30] Li P , Wang Y, Wang H et al Distance encoding: design provably more powerful neural networks for graph representation learning. Adv Neural Inform Proc Syst2020b;33:4465–78.

[btad241-B31] Lim T , Schaffner T, Crovella MA. multi-species functional embedding integrating sequence and network structure. In: *Research in Computational Molecular Biology–22nd Annual International Conference, RECOMB*. Berlin, Germany: Springer, 2018, 263–265.

[btad241-B32] Luo Y , Zhao X, Zhou J et al A network integration approach for drug-target interaction prediction and computational drug repositioning from heterogeneous information. Nat Commun 2017;8:1–13. 28924171 10.1038/s41467-017-00680-8 PMC5603535

[btad241-B33] Malod-Dognin N , Pržulj N. L-graal: lagrangian graphlet-based network aligner. Bioinformatics 2015;31:2182–9. 25725498 10.1093/bioinformatics/btv130 PMC4481854

[btad241-B34] Mamano N , Hayes WB. Sana: simulated annealing far outperforms many other search algorithms for biological network alignment. Bioinformatics 2017;33:2156–64. 28203713 10.1093/bioinformatics/btx090

[btad241-B35] Meng L , Striegel A, Milenković T et al Local versus global biological network alignment. Bioinformatics 2016;32:3155–64. 27357169 10.1093/bioinformatics/btw348 PMC5048063

[btad241-B36] Milenković T , Pržulj N. Uncovering biological network function via graphlet degree signatures. Cancer Inform 2008;6:CIN.S680. PMC2623288 19259413

[btad241-B37] Milenković T , Ng WL, Hayes W et al Optimal network alignment with graphlet degree vectors. Cancer Inform 2010;9:CIN.S4744. 10.4137/cin.s4744 PMC2901631 20628593

[btad241-B38] Nair V , Hinton GE. Rectified linear units improve restricted Boltzmann machines. In: *Proceedings of the 27th international conference on machine learning (ICML-10)*, 2010. pp. 807–14.

[btad241-B39] O'Neil NJ , Bailey ML, Hieter P. Synthetic lethality and cancer. Nat Rev Genet 2017;18:613–23. 28649135 10.1038/nrg.2017.47

[btad241-B40] Park CY , Wong AK, Greene CS et al Functional knowledge transfer for high-accuracy prediction of under-studied biological processes. PLoS Comput Biol 2013;9:e1002957. 23516347 10.1371/journal.pcbi.1002957 PMC3597527

[btad241-B41] Patro R , Kingsford C. Global network alignment using multiscale spectral signatures. Bioinformatics 2012;28:3105–14. 23047556 10.1093/bioinformatics/bts592 PMC3509496

[btad241-B42] Perozzi B , Al-Rfou R, Skiena S. Deepwalk: Online learning of social representations. In: *Proceedings of the 20th ACM SIGKDD International Conference on Knowledge Discovery and Data Mining*, 2014. pp. 701–10.

[btad241-B43] Qiu J , Dong Y, Ma H et al Network embedding as matrix factorization: unifying deepwalk, line, pte, and node2vec. In: *Proceedings of the Eleventh ACM International Conference on Web Search and Data Mining*, 2018. pp. 459–67.

[btad241-B44] Saraph V , Milenković T. Magna: maximizing accuracy in global network alignment. Bioinformatics 2014;30:2931–40. 25015987 10.1093/bioinformatics/btu409

[btad241-B45] Sarlin PE , DeTone D, Malisiewicz T et al Superglue: Learning feature matching with graph neural networks. In: *Proceedings of the IEEE/CVF Conference on Computer Vision and Pattern Recognition*, 2020. pp. 4938–47.

[btad241-B46] Singh R , Xu J, Berger B et al Global alignment of multiple protein interaction networks with application to functional orthology detection. Proc Natl Acad Sci USA 2008;105:12763–8. 18725631 10.1073/pnas.0806627105 PMC2522262

[btad241-B47] Stark C , Breitkreutz B-J, Reguly T et al Biogrid: a general repository for interaction datasets. Nucleic Acids Res 2006;34:D535–9. 16381927 10.1093/nar/gkj109 PMC1347471

[btad241-B48] Steinegger M , Söding J. Mmseqs2 enables sensitive protein sequence searching for the analysis of massive data sets. Nat Biotechnol 2017;35:1026–8. 29035372 10.1038/nbt.3988

[btad241-B49] Ullmann JR. An algorithm for subgraph isomorphism. J ACM (JACM) 1976;23:31–42. 10.1145/321921.321925.

[btad241-B50] Vijayan V , Milenković T. Multiple network alignment via multimagna. IEEE/ACM Trans Comput Biol Bioinform 2017;15:1669–82. 28829315 10.1109/TCBB.2017.2740381

[btad241-B51] Vijayan V , Milenković T. Aligning dynamic networks with dynawave. Bioinformatics 2018;34:1795–8. 29300873 10.1093/bioinformatics/btx841

[btad241-B52] Xu K , Hu W, Leskovec J et al How powerful are graph neural networks? *arXiv preprint arXiv:1810.00826*. 2018.

[btad241-B53] Zafarani R , Liu H. Connecting users across social media sites: a behavioral-modeling approach. In: *Proceedings of the 19th ACM SIGKDD International Conference on Knowledge Discovery and Data Mining*, 2013. pp. 41–9.

[btad241-B54] Zhao L , Akoglu L. Pairnorm: tackling oversmoothing in gnns. *arXiv preprint arXiv:1909.12223.* 2019.

[btad241-B55] Zhu H , Xie R, Liu Z et al Iterative entity alignment via knowledge embeddings. In: *Proceedings of the International Joint Conference on Artificial Intelligence (IJCAI),* 2017. pp. 4258–64.

