# Supplementary material for: Supervised biological network alignment with graph neural networks
Source: Bioinformatics. 2023 Jun 30;39(Suppl 1):i465–74. doi: 10.1093/bioinformatics/btad241 (PMC10311300; doi:10.1093/bioinformatics/btad241)
Supplement: btad241_Supplementary_Data [file btad241_supplementary_data.pdf]

1 Supplementary Information

1.1 Experimental setup

For network alignment prediction, GraNA was trained on the training set, tuned on the valid set, and evaluated on the test set along with other NA baselines, where the split ratio is 70/10/20. For each dataset, data split was performed 5 times with 5 different seeds.

In the context of NA for biological networks, many different evaluation metrics have been proposed, and they often focus on different aspects of network alignment prediction. Ma and Liao (2020) categorized some of the most commonly used metrics into two types: biological evaluation and topological evaluation. Apart from the metrics summarized by Ma and Liao (2020), Fan *et al.* (2019); Li *et al.* (2022) also used AUPRC and AUROC for evaluating the predicted network alignment. In this work, we selected metrics following Singh *et al.* (2008); Chindelevitch *et al.* (2013); Fan *et al.* (2019); Li *et al.* (2022) and included AUROC, AUPRC, Jaccard index (also known as Gene Ontology Consistency), and functional coherence (FC) as metrics. As the prediction of GraNA is a many-to-many mapping between the across-species proteins, we cannot directly leverage a particular set of metrics used in previous studies Saraph and Milenković (2014); Vijayan and Milenković (2017), such as Edge Correctness (EC) and Node Correctness (NC), which are based on the assumption that the mapping is one-to-one and require non-trivial modification for our purpose.

For protein function prediction, we chose the Jaccard index and functional coherence of the top 200 predicted node pairs given for each train/test split (1000 pairs in total after combining the top pairs from five runs). For a fair comparison, we filtered anchor links that coincide with positive test pairs from our training data. Jaccard index describes how similar two proteins are in terms of function, as it is calculated as $|S_1 \cap S_2|/|S_1 \cup S_2|$, where S_1, S_2 represent respectively the set of GO terms the two nodes are annotated with. Following previous studies (Singh *et al.*, 2008; Chindelevitch *et al.*, 2013), we define functional coherence as follows. GO terms were first mapped to a standardized GO set. Within this set, all GO terms are at a distance of 5 to the root of the GO hierarchy, and any GO terms with a distance less than 5 to the root are dropped. We measured the distance only by considering the relations *is a* and *part of* in Biological Process (BP) of the GO, and we retrieved the ancestor information of each GO term through the QuickGO REST API (Binns *et al.*, 2009). This design aimed to avoid evaluating functional similarity at different levels of the Gene Ontology graph. For each protein pair (x, y), functional coherence is defined as $|S_x \cap S_y|/|S_x \cup S_y|$, whereas S_x, S_y represent the sets of standardized GO terms with protein x, y respectively. Using Jaccard index and functional coherence, we are able to quantify the proportion of functional knowledge that is successfully transferred from the network alignment established by GraNA.

1.2 Baselines

In experiments, we compare GraNA with several existing NA methods. For a fair comparison, all baseline methods were trained, if needed, and evaluated on the same data as GraNA. Specifically, for baselines that require anchor links, we used the same ortholog anchor links that GraNA uses. Default parameters were used for all baselines.

For unsupervised NA method, we included IsoRank (Singh *et al.*, 2008), MMseqs2 (Steinegger and Söding, 2017), MUNK (Fan *et al.*, 2019), and ETNA (Li *et al.*, 2022). MMseqs2 is a tool for calculating sequence similarity and clustering proteins based on their sequences. We included it as a baseline method for assessing the relatedness of sequence similarity to functional similarity. IsoRank is an unsupervised multi-network alignment method, which is based on the intuition that functionally similar proteins have similar sequences and neighborhood topologies. The alignment of networks is formulated as an eigenvalue problem. IsoRank was originally designed to align orthologous pairs using sequence similarity as anchor links. MUNK, linking two PPIs via orthologs, uses matrix factorization to create a functional embedding in a way that proteins from different species are embedded in the same space. Then, a score matrix is calculated between two species, which can be used for network alignment prediction. ETNA is the state-of-the-art unsupervised NA method. It first learns representations for proteins from the PPIs via autoencoder and then applies a cross-training mechanism using orthologs to align the embeddings from two species. For the supervised NA method, we included TARA-TS and TARA++ (Gu and Milenković, 2021). From the three versions of TARA-TS (graphlet (Milenković and Pržulj, 2008), node2vec (Grover and Leskovec, 2016), metapath2vec (Dong *et al.*, 2017)), we chose the version based on node2vec as it showed the best performance among the three as shown in their experiments. Regarding TARA++, for the protein function prediction evaluation framework (Meng *et al.*, 2016), we implemented TARA++ according to its original definition, which is the intersection of TARA and TARA-TS predictions. For network alignment prediction, we had to make a tweak on TARA++: in the TARA++ paper, TARA++ was developed for the function prediction task but not for the network alignment task. Therefore, we adapted TARA++ to the network alignment prediction to compare with GraNA – we first ran TARA and TARA-TS to obtain the predicted probability (produced by the logistic regression classifier) that a given protein pair shares at least one GO term and then we took the average to TARA's and TARA-TS's predicted probabilities. The averaged probability was used as the prediction of TARA++. The average operation here followed the same idea of the intersection operation in the original TARA++ for function prediction, which took the consensus predictions of TARA and TARA-TS. In addition to the average, we have tried combining TARA and TARA-TS by taking their minimum or maximum predicted probability for network alignment, and the results were similar. Using this approach, we were able to compare TARA++ to other methods in our network alignment benchmark.

1.3 Hyperparameters

The hyperparameters in GraNA include the total number of epochs, batch size, learning rate, hidden dimension, number of graph convolution blocks, and graph convolution type. We comprehensively tested the robustness of GraNA against different hyperparameter settings. The search space of hyperparameters for training GraNA was shown in Table S3. For each train/valid/test split, GraNA was first trained on the training set and then validated on the validation set. We chose the final combination of hyperparameters for training GraNA based on GraNA's performances (AUROC and AUPRC) on the validation set. To avoid an exponential number of combinations of hyperparameters that would make the grid search infeasible, we fixed the values of other hyperparameters when tuning one specific hyperparameter.

We evaluated four different types of graph convolution layer: GCN (Kipf and Welling, 2016), SAGE (Hamilton *et al.*, 2017), GAT (Veličković *et al.*, 2017), and GEN (Li *et al.*, 2020). The four architectures differ from each other mainly in their neighborhood information aggregation mechanisms. GCN aggregates neighborhood information in a weighted mean manner based on node degrees and edge weights from the normalized Laplacian matrix. SAGE, in comparison, takes a mean over neighborhood node features for constructing the message for one node. GAT employs the attention mechanism for aggregating node features, whereas GEN is the layer we used in GraNA, and it aggregates neighborhood information through a softmax function.

Raw results averaged on five independent train/valid split for each hyperparameter setting for alignment between *S. cerevisiae* and *S. pombe* were shown in Figure S9. We observed that GraNA was robust to hyperparameters. Given the results of hyperparameter tuning and the computational resources available to us, we built a total of 7 graph convolution blocks, each with a hidden dimension of 128 and a convolution type of GEN (Li *et al.*, 2020), for GraNA. During training, we used the Adam optimizer with an initial learning rate of 0.001 and a weight decay of 5e-4, and we set the batch size to be 2^{16}. We trained GraNA for a maximum of 200 epochs. GraNA was trained on a single NVIDIA A40 GPU card. The running time analyses of GraNA and baseline methods (TARA, TARA-TS, ETNA) are provided in Table S6.

2 Supplementary Tables

Table S1. The number of nodes and edges in the PPI network of each species. Abbreviations: sce: *S. cerevisiae*, spo: *S. pombe*, hsa: *H. sapiens*, mmu: *M. Musculus*, cel: *C. elegans*, and dme: *D. melanogaster*.

Type	sce	spo	hsa	mmu	cel	dme
Nodes	5,669	2,334	17,120	7,762	4,439	7711
Edges	110,776	10,525	418,512	47,833	18,301	49,769

Table S2. The number of anchor links (orthologs and sequence similarity) and protein pairs sharing function in each pair of PPI networks. *Orth only*: anchor links that were included only as orthologs; *Both orth and seq*: anchor links that were both included as orthologs and sequence similarity relationships; *Seq only*: anchor links that were included only as sequence similarity; *Pairs sharing func*: cross-species protein pairs that share at least one function. Species abbreviations are identical to Table S1.

Type	sce-spo	hsa-sce	hsa-mmu	hsa-cel	hsa-dme
Orthologs	1,485	2,221	10,819	2,561	4,603
Seq similarity	8,324	37,711	191,172	23,419	40,828
Orth only	555	878	3,208	1,400	2,963
Both orth and seq	930	1,343	7,611	1,161	1,640
Seq only	7,394	36,368	183,561	22,258	39,188
Pairs sharing func	195,519	1,021,948	1,938,820	327,907	1,090,256

Table S3. The search space of hyperparameters for training GraNA. GraNA is trained on train set and validated on valid set. The final combination of hyperparameters is determined based on GraNA's performance on the valid set.

Hyperparameter	Range
Epochs	[50,100,200,300]
Batch size	[2^{13}, 2^{14}, 2^{15}, 2^{16}, 2^{17}]
Learning rate	[0.0001, 0.001, 0.01]
Hidden dimension	[32, 64, 128, 256]
Block number	[1, 3, 5, 7, 9]
Convolution type	[GCN, SAGE, GAT, GEN]

Table S4. AUROC of GraNA and baseline methods for predicting network alignment across species. For each dataset, we reported the AUROC values averaged over five independent train/test data splits. The abbreviations are identical to Table S1.

Method	sce-spo	hsa-sce	hsa-mmu	hsa-cel	hsa-dme
MMseqs2	0.5057	0.5095	0.5102	0.5117	0.5101
IsoRank	0.5650	0.5179	0.5104	0.5143	0.5129
MUNK-f	0.5644	0.5819	0.5372	0.5111	0.5641
MUNK-b	0.5566	0.5772	0.5288	0.5079	0.5576
TARA-TS	0.6241	0.6384	0.6495	0.5848	0.6346
TARA++	0.6270	0.6311	0.6533	0.5921	0.6372
ETNA	0.7045	0.6631	0.5805	0.5784	0.5891
GraNA-o	0.7707	0.6944	0.6568	0.6174	0.6367
GraNA-s	0.7681	0.6952	0.6473	0.6000	0.6287
GraNA	**0.7865**	**0.7165**	**0.6755**	**0.6335**	**0.6506**

Table S5. AUPRC of GraNA and baseline methods for predicting network alignment across species. For each dataset, we reported the AUPRC values averaged over five independent train/test data splits. The abbreviations are identical to Table S1.

Method	sce-spo	hsa-sce	hsa-mmu	hsa-cel	hsa-dme
MMseqs2	0.0598	0.0547	0.0683	0.0635	0.0572
IsoRank	0.0770	0.0476	0.0628	0.0558	0.0512
MUNK-f	0.0748	0.0562	0.0675	0.0569	0.0578
MUNK-b	0.0740	0.0556	0.0661	0.0559	0.0564
TARA-TS	0.1019	0.0841	0.1140	0.0720	0.0842
TARA++	0.0927	0.0756	0.1168	0.0783	0.0861
ETNA	0.1832	0.1053	0.0914	0.0720	0.0706
GraNA-o	0.2635	0.1258	0.1320	0.0931	0.0956
GraNA-s	0.2670	0.1336	0.1359	0.0970	0.1010
GraNA	**0.2892**	**0.1511**	**0.1518**	**0.1078**	**0.1120**

Table S6. Running time analysis of GraNA and baseline methods TARA, TARA-TS, and ETNA. The time needed for building topological features and training model were reported in minutes. Inference time could be neglected compared to feature-building and model-training time. The abbreviations are identical to Table S1.

Method	Time	sce-spo	hsa-sce	hsa-mmu	hsa-cel	hsa-dme
TARA	feature	47	205	150	137	162
	train	<1	1	2	<1	1
TARA-TS	feature	<1	1	1	1	1
	train	<1	1	2	<1	1
ETNA	feature	<1	7	7	6	7
	train	<1	<1	<1	<1	<1
GraNA	feature	<1	7	7	6	7
	train	9	76	117	20	75

3 Supplementary Figures

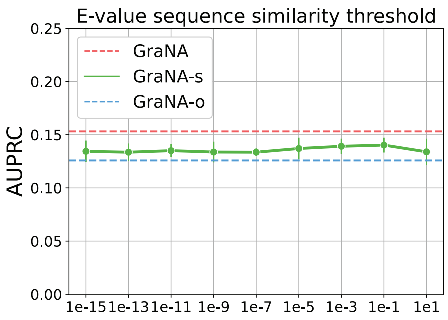

Fig. S1. Impacts of E-value cutoff values used to identify sequence-similar protein pairs as anchor links. In GraNA, sequence similarity relationships are used as one type of anchor link. These sequence-similar protein pairs were identified by performing a sequence similarity search by MMseqs2 and selecting those pairs with an E-value smaller than a cutoff. We trained a GraNA variant that only used sequence similarity as anchor links (labeled as GraNA-s) and evaluated its AUPRC score of aligning the PPI networks of *H. sapiens* and *S. cerevisiae* when different E-value cutoffs were used. For reference, the AUPRC scores of GraNA-o and GraNA were shown. Since GraNA-o did not include sequence similarity as anchor links and GraNA used the default E-value cutoff of 10^{-7}, their AUPRC scores were constant values in the figure.

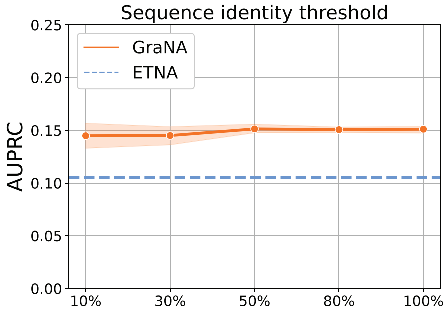

Fig. S2. AUPRC of GraNA on data splits with different sequence identity thresholds. To validate GraNA's effectiveness, we evaluate GraNA using harder data splits, which require that the train split and the test split are dissimilar in sequences. In practice, we fix the training sets and only filter test sets. Using MMseqs2 (Steinegger and Söding, 2017) to search the proteins in the test set that are under the sequence identity threshold, we constitute new test sets for each threshold. We select sequence identity thresholds 10%, 30%, 50%, 80%, and 100% (the original test split) and evaluate GraNA's performance for each threshold on five independent data splits for *H. sapiens* and *S. cerevisiae*.

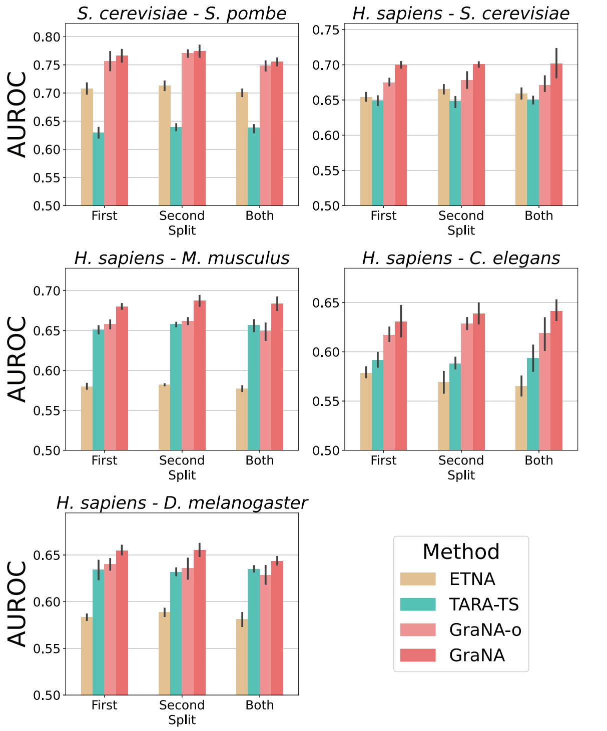

Fig. S3. AUROC of network alignment prediction on sequence identity-based data splits. To further validate GraNA's effectiveness under difficult data splits, we compared GraNA with the best unsupervised and supervised baselines (ETNA and TARA-TS) and a variant of GraNA (GraNA-o), that only uses orthologs as anchor links, on data splits that ensured proteins from the train split and the test split are dissimilar in terms of their sequence identity. Compared to the train/test splits in Fig. 2 where test proteins are ensured to not appear in the training set, here we create several more challenging train/test splits such that for the chosen species (the first species, the second species, or both species), its proteins in the test split must have sequence identity lower than 30% to its proteins in the train split. In our experiments, we iteratively sampled proteins and added those proteins together with their sequence-similar proteins (above 30% sequence identity) to the test set. The sequence identity is calculated by BLASTp (Camacho et al., 2009).

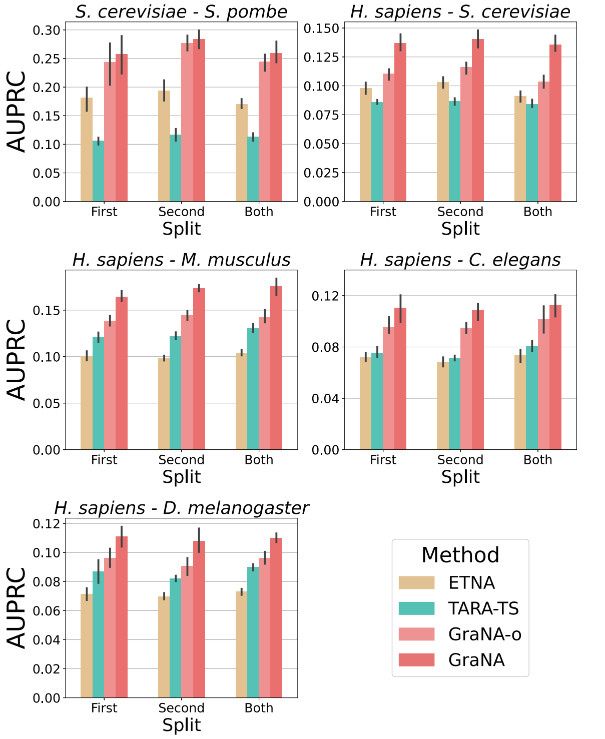

Fig. S4. AUPRC of network alignment prediction on sequence identity-based data splits. To further validate GraNA's effectiveness under difficult data splits, we compared GraNA with the best unsupervised and supervised baselines (ETNA and TARA-TS) and a variant of GraNA (GraNA-o), that only uses orthologs as anchor links, on data splits that ensured proteins from the train split and the test split are dissimilar in terms of their sequence identity. Compared to the train/test splits in Fig. 2 where test proteins are ensured to not appear in the training set, here we create several more challenging train/test splits such that for the chosen species (the first species, the second species, or both species), its proteins in the test split must have sequence identity lower than 30% to its proteins in the train split. In our experiments, we iteratively sampled proteins and added those proteins together with their sequence-similar proteins (above 30% sequence identity) to the test set. The sequence identity is calculated by BLASTp (Camacho et al., 2009).

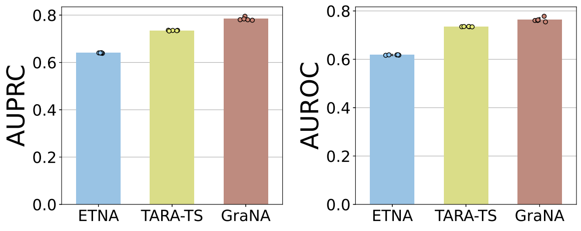

Fig. S5. Network alignment performance on predicting newly discovered alignments between *H. sapiens-M. Musculus* based on known alignments. To further demonstrate GraNA's potential for application, we compared GraNA with the best unsupervised and supervised baselines (ETNA and TARA-TS) on predicting the newly discovered alignments from GO (Consortium, 2004) (2022-12-04) that are not included in GO (Consortium, 2004) (2018-07-02). Following the method of generating the alignments in the benchmark dataset, we first create a slim set of GO terms from GO (2018-07-02) and then use it to generate new alignments in GO (2022-12-04), which contains 48% more functionally similar pairs. Supervised methods are trained on the supervision from 2018. All methods are evaluated on the dataset that includes all newly discovered alignments as positive samples and negative samples downsampled to an equal amount of positive samples. Experiments were repeated using five random seeds for negative sampling.

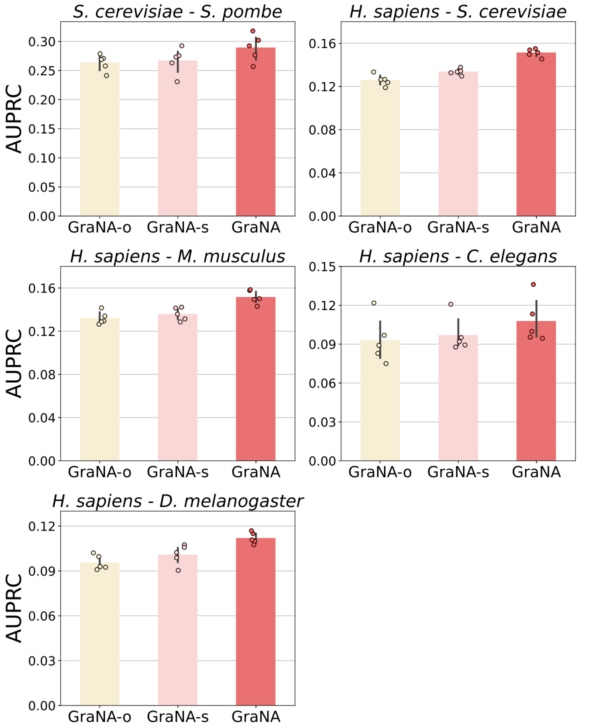

Fig. S6. Network alignment performance of GraNA using different anchor links. We evaluated the performances of GraNA using only orthologs, only sequence similarity, and both orthologs and sequence similarity as anchor links for network alignment. Five pairs of PPIs (*S. cerevisiae-S. pombe*, *H. sapiens-S. cerevisiae*, *H. sapiens-M. Musculus*, *H. sapiens-C. elegans*, *H. sapiens-D. melanogaster*) are used for evaluation. AUPRC of five independent train/test data splits were reported.

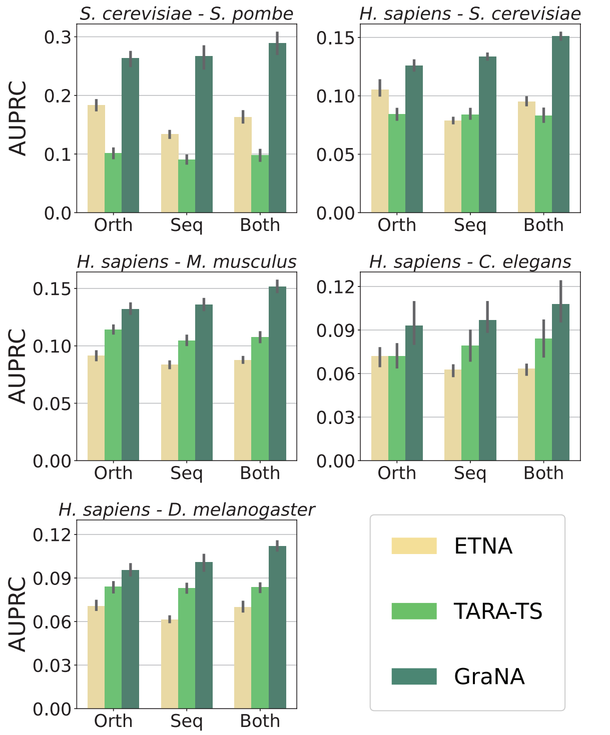

Fig. S7. Evaluation of the ability to integrate heterogeneous anchor links. To validate GraNA's ability to leverage orthology information and sequence similarity information at the same time, we compared GraNA with two of the best baselines, TARA-TS and ETNA, for network alignment using different anchor links. We used either orthologs, sequence similarity, or both orthologs and sequence similarity as anchor links for aligning five pairs of PPIs (*S. cerevisiae-S. pombe*, *H. sapiens-S. cerevisiae*, *H. sapiens-M. Musculus*, *H. sapiens-C. elegans*, *H. sapiens-D. melanogaster*), on five independent data splits. Abbreviations: orth: orthologs; seq: sequence similarity.

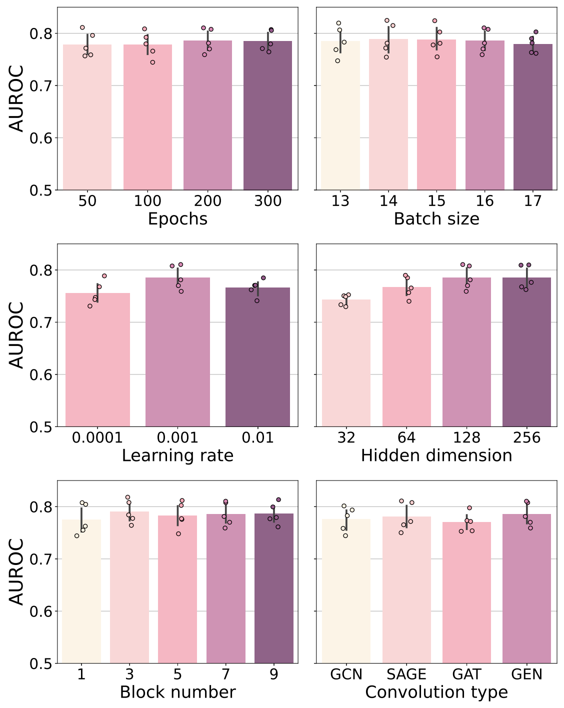

Fig. S9. AUROC of GraNA for predicting the alignment between *S. cerevisiae* and *S. pombe* averaged over five independent data splits on valid set. While we are evaluating one type of hyperparameter, the other hyperparameters remain fixed. We evaluated in total 6 types of hyperparameters, including the total number of epochs, batch size, learning rate, hidden dimension, block number, and convolution type.

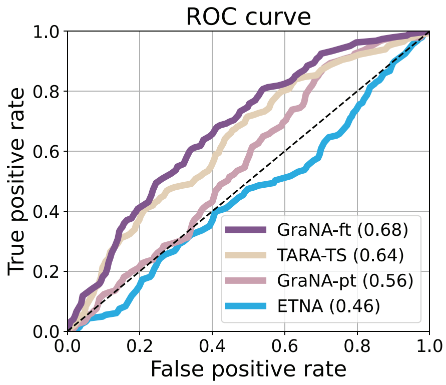

Fig. S8. ROC curve of GraNA and baselines in the case study. We further included TARA-TS for comparison in predicting the replaceability of human genes with their yeast orthologs. TARA-TS is trained and evaluated on the dataset of experimental results by Kachroo et al. (Kachroo et al., 2015) via five-fold cross-validation.

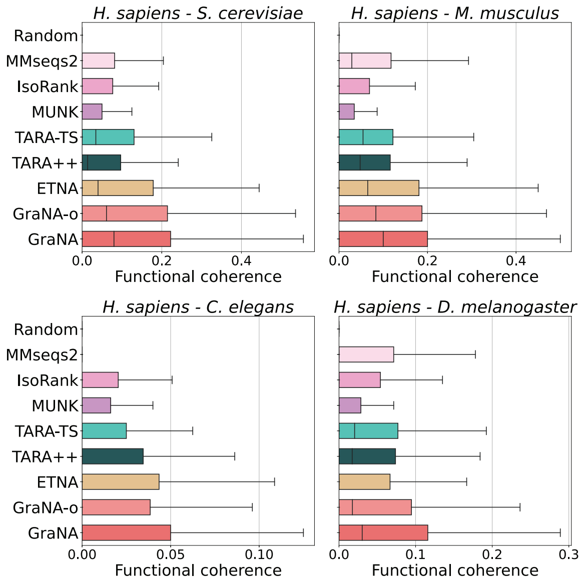

Fig. S10. Functional coherence (FC) based on the network alignments produced by each method for four pairs of species (*H. sapiens-S. cerevisiae*, *H. sapiens-M. Musculus*, *H. sapiens-C. elegans*, *H. sapiens-D. melanogaster*). We chose the top 5,000 ranked protein pairs and transferred all the functional annotations of one protein in an aligned pair to predict the other protein's function. The accuracy of the function prediction was evaluated by calculating the FC between the sets of the two aligned proteins. Unlike Jaccard index, FC only focuses on standardized GO terms (at a distance 5 to the root of the GO root) to avoid bias caused by terms from different levels of the GO hierarchy. Box plots showed the distribution of the FC of the top 5,000 aligned pairs for each method on five NA tasks under five random seeds.

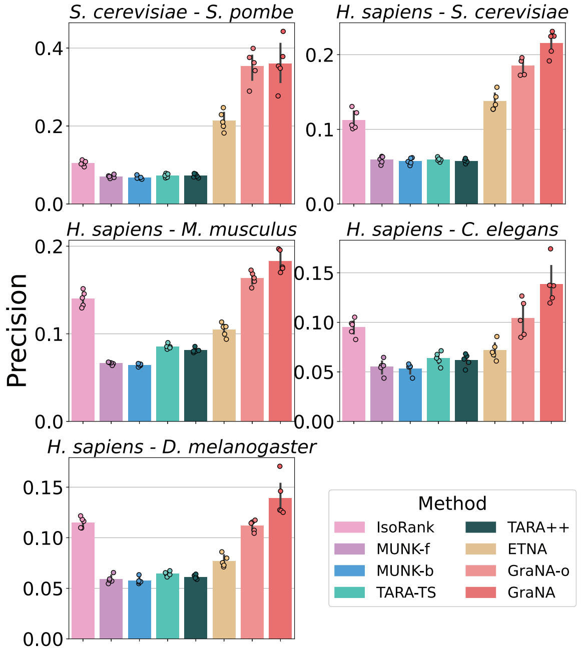

Fig. S11. Precision of network alignment prediction. GraNA and other baselines were evaluated for aligning functionally similar proteins across five pairs of species, and we used precision as the metric. For GraNA's predictions, we first selected the probability threshold maximizing the f1 score on the valid set and used this threshold to make final alignment predictions on the test set. GraNA-o is a variant of GraNA that only uses orthologs as anchor links whereas GraNA refers to the full model that uses both orthologs and sequence similarity as anchor links. As MUNK is not a bidirectional NA method, the performances of its forward and backward predictions were shown separately as MUNK-f and MUNK-b. Performances were evaluated using five independent train/test data splits.

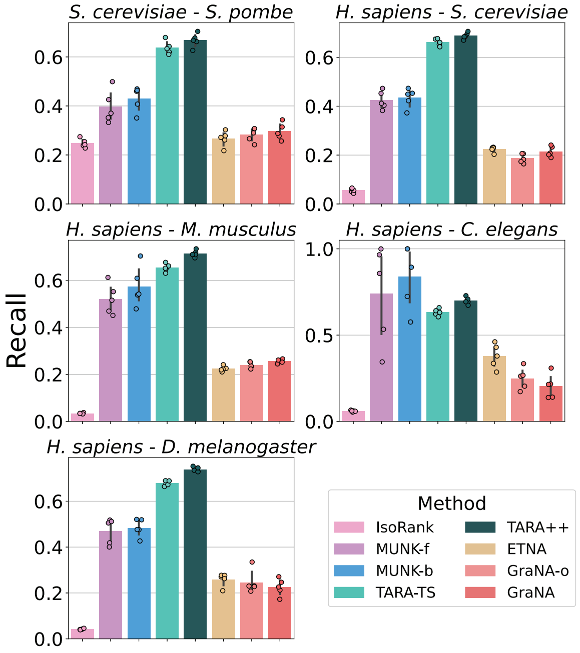

Fig. S12. Recall of network alignment prediction. GraNA and other baselines were evaluated for aligning functionally similar proteins across five pairs of species, and we used recall as the metric. For GraNA's predictions, we first selected the probability threshold maximizing the f1 score on the valid set and used this threshold to make final alignment predictions on the test set. GraNA-o is a variant of GraNA that only uses orthologs as anchor links whereas GraNA refers to the full model that uses both orthologs and sequence similarity as anchor links. As MUNK is not a bidirectional NA method, the performances of its forward and backward predictions were shown separately as MUNK-f and MUNK-b. Performances were evaluated using five independent train/test data splits.

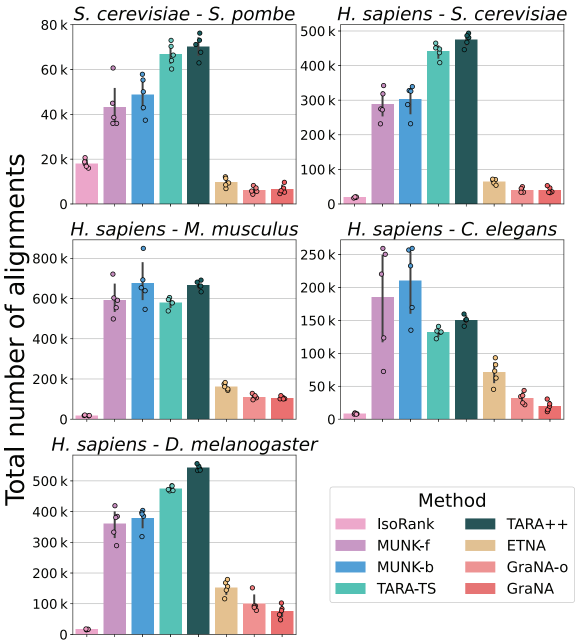

Fig. S13. Total number of predicted network alignments. GraNA and other baselines were evaluated for aligning functionally similar proteins across five pairs of species, and we reported the total number of network alignments predicted by each method. For GraNA's predictions, we first selected the probability threshold maximizing the f1 score on the valid set and used this threshold to make final alignment predictions on the test set. GraNA-o is a variant of GraNA that only uses orthologs as anchor links whereas GraNA refers to the full model that uses both orthologs and sequence similarity as anchor links. As MUNK is not a bidirectional NA method, the performances of its forward and backward predictions were shown separately as MUNK-f and MUNK-b. Performances were evaluated using five independent train/test data splits.

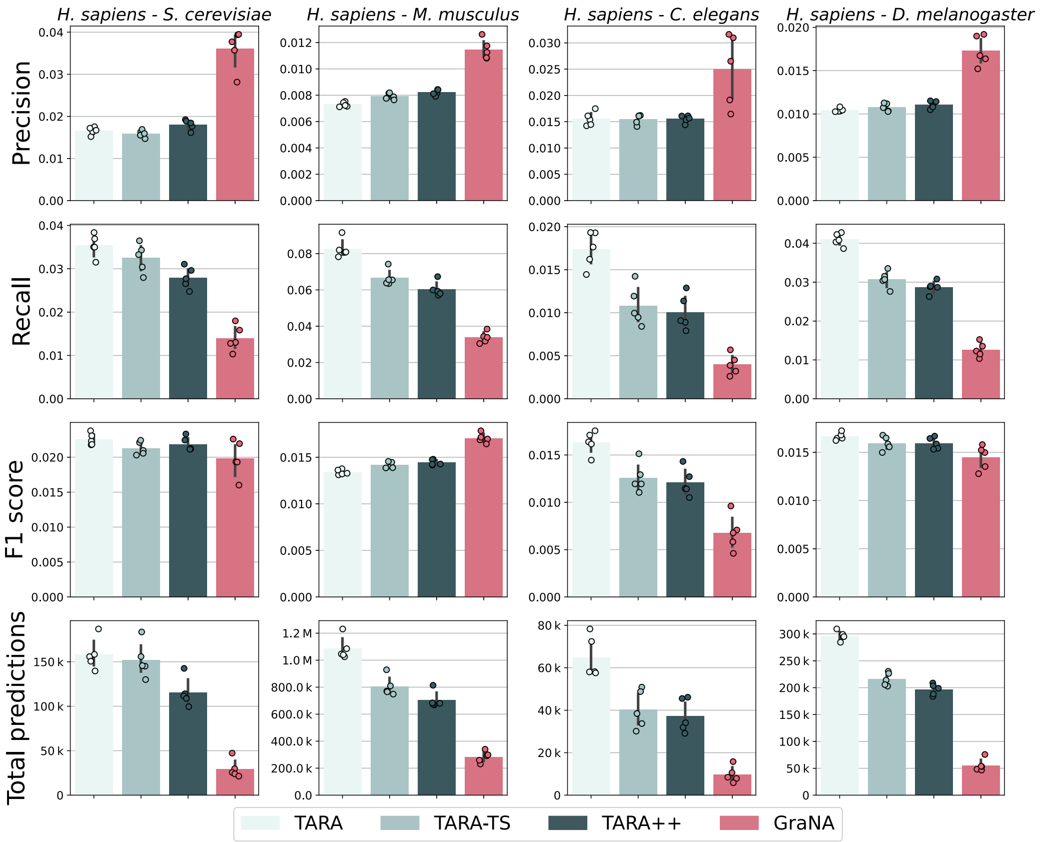

Fig. S14. Performances of protein function prediction. We evaluated TARA, TARA-TS, TARA++, and GraNA in the context of cross-species protein function prediction in an established protein function prediction framework (Meng et al., 2016), and we used precision, recall, F1 score, and total number of predictions (protein-GO pairs) as metrics. The evaluation framework starts by performing network alignment prediction on a test set of protein pairs, and then it evaluates the functional predictions made based on the predicted network alignment via statistical tests. Consistent with other experiments in our manuscript, we only evaluated the methods on pairs of proteins that both have at least one alignment. As the data was unbalanced in the test set, we subsampled negative pairs of proteins to the number of positive pairs of proteins to construct a balanced test set. We restricted the function prediction only for GO terms from the slim set to avoid transferring general GO terms such as Biological Process. TARA++ prediction was the overlap of the predictions of TARA and TARA-TS. Performances were evaluated using five independent train/test data splits.

References

Binns, D. *et al.* (2009). Quickgo: a web-based tool for gene ontology searching. *Bioinformatics*, **25**(22), 3045–3046.

Camacho, C. *et al.* (2009). Blast+: architecture and applications. *BMC bioinformatics*, **10**, 1–9.

Chindelevitch, L. *et al.* (2013). Optimizing a global alignment of protein interaction networks. *Bioinformatics*, **29**(21), 2765–2773.

Consortium, G. O. (2004). The gene ontology (go) database and informatics resource. *Nucleic acids research*, **32**(suppl_1), D258–D261.

Dong, Y. *et al.* (2017). metapath2vec: Scalable representation learning for heterogeneous networks. In *Proceedings of the 23rd ACM SIGKDD international conference on knowledge discovery and data mining*, pages 135–144.

Fan, J. *et al.* (2019). Functional protein representations from biological networks enable diverse cross-species inference. *Nucleic acids research*, **47**(9), e51–e51.

Grover, A. and Leskovec, J. (2016). node2vec: Scalable feature learning for networks. In *Proceedings of the 22nd ACM SIGKDD international conference on Knowledge discovery and data mining*, pages 855–864.

Gu, S. and Milenković, T. (2021). Data-driven biological network alignment that uses topological, sequence, and functional information. *BMC bioinformatics*, **22**(1), 1–24.

Hamilton, W. *et al.* (2017). Inductive representation learning on large graphs. *Advances in neural information processing systems*, **30**.

Kachroo, A. H. *et al.* (2015). Systematic humanization of yeast genes reveals conserved functions and genetic modularity. *Science*, **348**(6237), 921–925.

Kipf, T. N. and Welling, M. (2016). Semi-supervised classification with graph convolutional networks. *arXiv preprint arXiv:1609.02907*.

Li, G. *et al.* (2020). Deepergcn: All you need to train deeper gcns. *arXiv*.

Li, L. *et al.* (2022). Joint embedding of biological networks for cross-species functional alignment. *bioRxiv*.

Ma, C.-Y. and Liao, C.-S. (2020). A review of protein–protein interaction network alignment: From pathway comparison to global alignment. *Computational and Structural Biotechnology Journal*, **18**, 2647–2656.

Meng, L. *et al.* (2016). Local versus global biological network alignment. *Bioinformatics*, **32**(20), 3155–3164.

Milenković, T. and Pržulj, N. (2008). Uncovering biological network function via graphlet degree signatures. *Cancer informatics*, **6**, CIN–S680.

Saraph, V. and Milenković, T. (2014). Magna: maximizing accuracy in global network alignment. *Bioinformatics*, **30**(20), 2931–2940.

Singh, R. *et al.* (2008). Global alignment of multiple protein interaction networks with application to functional orthology detection. *Proceedings of the National Academy of Sciences*, **105**(35), 12763–12768.

Steinegger, M. and Söding, J. (2017). Mmseqs2 enables sensitive protein sequence searching for the analysis of massive data sets. *Nature biotechnology*, **35**(11), 1026–1028.

Veličković, P. *et al.* (2017). Graph attention networks. *arXiv preprint arXiv:1710.10903*.

Vijayan, V. and Milenković, T. (2017). Multiple network alignment via multimagna++. *IEEE/ACM transactions on computational biology and bioinformatics*, **15**(5), 1669–1682.
